# Anti-Oxidant Multi-Functionalized Materials: Strontium-Substituted Monetite and Brushite as Delivery Systems for Curcumin

**DOI:** 10.3390/pharmaceutics15051344

**Published:** 2023-04-27

**Authors:** Francesca Silingardi, Stefania Pagani, Alessandro Gambardella, Gianluca Giavaresi, Adriana Bigi, Elisa Boanini

**Affiliations:** 1 Department of Chemistry ‘‘Giacomo Ciamician”, University of Bologna, Via Selmi 2, 40126 Bologna, Italy; 2 Complex Structure Surgical Sciences and Technologies, IRCCS Istituto Ortopedico Rizzoli, Bologna, Via di Barbiano 1/10, 40136 Bologna, Italy

**Keywords:** calcium phosphates, radical scavenging activity, co-cultures

## Abstract

Curcumin has numerous biological activities and pharmaceutical applications related to its ability to inhibit reactive oxygen species. Herein, strontium-substituted monetite (SrDCPA) and strontium-substituted brushite (SrDCPD) were synthesized and further functionalized with curcumin with the aim to develop materials that combine the anti-oxidant properties of the polyphenol, the beneficial role of strontium toward bone tissue, and the bioactivity of calcium phosphates. Adsorption from hydroalcoholic solution increases with time and curcumin concentration, up to about 5–6 wt%, without affecting the crystal structure, morphology, and mechanical response of the substrates. The multi-functionalized substrates exhibit a relevant radical scavenging activity and a sustained release in phosphate buffer. Cell viability, morphology, and expression of the most representative genes were tested for osteoclast seeded in direct contact with the materials and for osteoblast/osteoclast co-cultures. The materials at relatively low curcumin content (2–3 wt%) maintain inhibitory effects on osteoclasts and support the colonization and viability of osteoblasts. The expressions of Alkaline Phosphatase (*ALPL*), collagen type I alpha 1 chain (*COL1A1*), and osteocalcin (*BGLAP*) suggest that curcumin reduces the osteoblast differentiation state but yields encouraging osteoprotegerin/receptor activator for the NFkB factor ligand (*OPG/RANKL*) ratio.

## 1. Introduction

Research on biomaterials for the substitution/repair of biological tissues shows an increasing interest in multi-functionalized materials, which are able to sum up the desirable beneficial effects of the different components [1]. As concerns of hard tissue disorders, a relevant part of the activity is addressed to the development of calcium orthophosphates (CaPs) functionalized with bioactive substances, including ions, macromolecules, drugs, and molecules displaying therapeutic properties [2].

In this work, strontium-substituted calcium phosphate materials are loaded with an anti-inflammatory natural molecule, namely curcumin [3].

Strontium is known to prevent the abnormal bone resorption characteristic of some diseases, such as osteoporosis [4]. Literature reports many in vitro and in vivo studies that highlight the role of strontium in the promotion of osteoblast proliferation and activity while inhibiting osteoclastogenesis [4,5,6,7].

Curcumin’s structure was identified in the early years of the previous century [8], but its use as Ayurvedic and Chinese medical agent, as well as spice, dates back to about 4000 years ago [9]. Curcumin, (1E,6E)-1,7-bis(4-hydroxy-3-methoxyphenyl)-1,6-heptadiene-3,5-dione, is one of the main curcuminoids present in the rhizome of *Curcuma Longa* [10]. This yellow pigment of relatively small molecular mass (368.67 g/mol) is a non-flavonoid polyphenol containing two aryl rings symmetrically connected to a β-diketone fraction [11]. Curcumin acts as a free radical scavenger and can regulate many biological processes thanks to its important antioxidant power, related to its capability to inhibit reactive oxygen species, including superoxide anions, hydroxyl radicals, peroxide, and nitrite radicals [12,13,14]. The reduction of the production of oxidation-derived free radicals suggests a beneficial action on bone tissue repair, as previously verified for other polyphenols [15].

Herein, two different CaPs, namely the anhydrous (CaHPO_4_) and dihydrated (CaHPO_4_·2H_2_O) dicalcium phosphate—commonly known as monetite (DCPA) and brushite (DCPD), respectively, are proposed as supports for curcumin. Both DCPA and DCPD were synthesized in the presence of strontium ions so as to obtain a partial substitution of strontium for calcium (of about 10 atom%).

The presence of brushite has been reported in several pathological calcifications [16,17], whereas neither brushite nor monetite have been found in physiologically calcified tissues. However, they demonstrated good osteoinductive and osteoconductive properties [18,19,20,21], which justifies their wide employment as components in a variety of biomaterials [17,18,22]. It was recently shown that the response of osteoblast and osteoclast cells co-cultured on these two phosphates is modulated by the presence of strontium inside their structures [7].

In the present work, the capability of strontium-substituted monetite (SrDCPA) and strontium-substituted brushite (SrDCPD) to adsorb curcumin has been studied. The characterization of the functionalized materials included their structural, morphological, and anti-oxidant properties, as well as their influence on bone cells.

## 2. Materials and Methods

### 2.1. Synthesis of Materials

Strontium-substituted DCPA and DCPD crystals (SrDCPA and SrDCPD) were obtained by direct synthesis using starting solutions containing 20 Sr atom% with respect to total cations, calculated as ([Sr^2+^/(Ca^2+^ + Sr^2+^)] × 100). All reagents were analytical grade (Carlo Erba Reagents, Milano, Italy).

In particular, SrDCPA crystals were obtained using 50 mL of an aqueous solution containing Ca(NO_3_)_2_∙4H_2_O (0.864 M) and Sr(NO_3_)_2_ (0.216 M) at 90 °C. A 50 mL solution containing (NH_4_)_2_HPO_4_ (0.65 M) was added dropwise (2 mL/min). After the addition, the solution was maintained at 90° and under stirring for 1 h, then the precipitate was centrifuged, washed with distilled water, and dried overnight at 37 °C.

SrDCPD crystals were prepared using 50 mL of an aqueous solution containing Ca(CH_3_COO)_2_∙H_2_O (0.16 M) and Sr(CH_3_COO)_2_∙½H_2_O (0.04 M) that were added dropwise (2 mL/min) to 150 mL of a solution containing Na_2_HPO_4_·12H_2_O (0.033 M) and NaH_2_PO_4_·H_2_O (0.033 M) whose pH was previously adjusted to five, adding glacial CH_3_COOH. During dropping and for the following 10 min, the solution was kept at 37 °C and under stirring (120 rpm); afterward, the precipitate was filtered, washed with distilled water, and dried overnight at 37 °C.

The content of Ca and Sr in the solid products was analyzed by ion chromatography (Dionex ICS-90, Dionex, Sunnyvale, CA, USA) by dissolving the solid powders in 0.1 M HCl. The calibration lines contained four calibration standards (Ca: 5, 20, 40, 60 mg/L; Sr: 2, 4, 10, 20 mg/L), prepared by the dilution of 1000 mg/L of calcium or strontium standard solutions.

For curcumin loading, 0.5 g of powder sample (SrDCPA or SrDCPD) were kept in a hydroalcoholic solution EtOH:H_2_O = 1:1 containing curcumin (Merck KGaA, Darmstadt, Germany) at different concentrations (3 mM, 4 mM, or 5 mM). Incubation was performed under stirring (300 rpm) for set periods of time (6 h and 72 h). Samples were then filtered and dried overnight at 37 °C.

The resulting samples were labeled as SrDCPAx_yh, or SrDCPDx_yh, where x is the concentration of curcumin solution and y is the time of incubation.

### 2.2. Physico-Chemical Characterization of Materials

#### 2.2.1. Preliminary Tests

For the determination of the curcumin content, about 10 mg of the sample were dissolved in 0.1 mL of HCl 6 M and then diluted in 10 mL H_2_O/EtOH 1:1. The analyzed sample was a 1/20 dilution of the first solution. UV-visible absorption spectra were collected with a Varian Cary50Bio instrument (λ = 430 nm). Each analysis was performed in triplicate.

X-ray diffraction (XRD) scans were acquired with a PANalytical X’Pert PRO diffractometer equipped with a Cu Kα source (λ = 1.5418 Å, 40 mA, 40 kV) and a fast X’Celerator detector in the Bragg−Brentano geometry. All patterns were collected in the 2θ range 3–60 2θ° for 100 s for each 0.1° step. The HighScore Plus program was used for phase identification and structural refinements (HighScore Plus software version 4.9, PANalytical B.V., Almelo, The Netherlands).

#### 2.2.2. Complete Tests

Based on the curcumin content and XRD results, the following studies were performed only on selected samples that have been labeled with shorter labels for the sake of clarity, as shown in Table 1.

Curcumin release tests were carried out on 40 mg of samples (SA3, SA5, SD3, and SD5) in 2 mL of PBS (pH = 7.4) at 37 °C. Each experiment was performed in triplicate. The release was monitored by collecting UV-vis absorption spectra on supernatant with a Varian Cary50Bio instrument (λ = 430 nm). The spectra were recorded at selected times (1, 3, 5, 7, and 14 days), and the solution was refreshed at each time.

Antioxidant activity was determined on the basis of curcumin’s ability to act as radical scavengers toward the 2,2-diphenyl-1-picrylhydrazyl free radical (DPPH•) (Sigma-Aldrich, Milano, Italy) [23]. Solutions of SA3, SA5, SD3, and SD5 were prepared by dissolving, with the lowest amount of HCl 6 M, the solid samples in MilliQ water/EtOH. Afterward, they were diluted, based on their known curcumin contents, in order to obtain 5 µM, 15 µM, and 30 µM of curcumin concentrations. Dilutions of pure curcumin, 5 µM, 15 µM, and 30 µM, were prepared and used as reference samples. For the assay, 5.4 mL of each solution (samples and references) were added to 0.6 mL of a 1 mM solution of DPPH• in EtOH. After 30 min of incubation at room temperature, in darkness, and under stirring, absorbance values were spectrophotometrically measured at 524 nm. The radical scavenging activity (RSA) was determined through the following equation: % RSA = [(A0 − Ax)/A0] × 100, where A0 is the absorbance of the control (containing DPPH• solution without curcumin), and Ax is the absorbance in the presence of pure curcumin (as reference) or of dissolved curcumin-containing samples.

In vitro tests were performed on disk-shaped samples (Ø = 6.0 mm). Each disk was prepared by pressing 40 mg of powder into cylindrical molds using a standard evacuable pellet die (Hellma Italia Srl, Milano, Italy) and sterilized using gamma rays (Cobalt-60) at a dose of 25 kGy.

Morphological investigation of the as-synthesized crystals and the disk-shaped samples surface was performed using a Zeiss Leo-1530 (Zeiss Microscopy, Milano, Italy) high-resolution scanning electron microscope (SEM) operating at 1 kV (InLens detector). The samples were sputter-coated with gold before observation.

Atomic Force Microscopy (AFM) measurements were performed by an NT-MDT (Moscow, Russia) system equipped with an upright optical microscope. NSG30 tips (NT-MDT, Moscow, Russia) with resonant frequency in the range of 140–390 kHz were used. All topographic images were taken at 256 × 256-pixel resolution and acquired in tapping mode. The cantilever stiffness k was in the range of 30–40 N/m. The cantilever deflection sensitivity was calibrated from a set of indentation curves previously obtained under a hard-contact regime on a clean and nanometrically flat silica slice (~80 GPa in stiffness); such procedure allows non-invasive calibration of a cantilever, i.e., without causing the damage of tips. Two-dimensional arrays of F-d curves (1000 points each) were acquired at several non-overlapped 5 × 5 µm^2^ areas on 20 × 20 grids. Before and after mapping, the tip integrity was checked via *z*-axis calibration on a TGS1 calibration grating (NT-MDT, Moscow, Russia; grid TGZ1 with height (21 ± 1) nm). From each F-d curve, the maximum penetration depth, h_max_, was extracted, and an elasticity index, 0 < η_el_ < 1, defined as the ratio between the areas under the unloading and loading curve, was calculated. For inelastic deformation, η_el_ = 0, while for purely elastic deformation, η_el_ = 1 [24]. Finally, the indentation modulus was extracted from each curve by using a Hertzian deformation model [25]. For calculations, Poisson’s ratios of 0.20 ± 0.02 and 0.26 ± 0.03 were assumed, respectively, for monetite and brushite.

### 2.3. Biological Assays

Biological tests were performed on disk-shaped samples of strontium-substituted monetite (SA) and brushite (SD) doped with curcumin 3% (SA3, SD3) or 5% (SA5, SD5).

#### 2.3.1. Cell Cultures

The present study was developed in two phases. Firstly, these biomaterials based on monetite or brushite partially substituted with strontium and doped with curcumin were tested by cultures of human primary osteoclast (OC). Subsequently, the biomaterials were tested by co-cultures consisting of OCs and human primary osteoblasts (NHOst).

OCs were obtained by mononuclear cells isolated from the peripheral human buffy coat of a healthy adult male donor (Ethic Committee -CE AVEC- approval n. 191/2019/Sper/IOR, 04/19) by density gradient centrifugation (Ficoll Histopaque 1077, Sigma Aldrich, St. Louis, MO, USA), as previously reported [1].

The obtained cells were seeded at the density of 1 × 10^6^ cells/cm^2^ in 24-wells plates in Dulbecco’s modified Eagle medium (DMEM, Sigma Aldrich) supplemented with 10% FCS and antibiotics (100 U/mL penicillin, 100 μg/mL streptomycin) and osteoclastogenic factors (macrophage colony-stimulating factor, MCSF, 25 ng/mL, and receptor activator for κB factor ligand, RANKL, 30 ng/mL, DMEM-OC) (Peprotech, Rocky Hill, NJ, USA). The plate was maintained for 7 days in standard conditions (37 °C ± 0.5 temperature, 5% CO_2_ ± 0.2, 95% humidity) to differentiate in osteoclasts (OC). After this time, each material was placed on the OC layer, and the cell was evaluated at 7 and 14 days (Figure S1-Phase 1). OCs without materials were used as the control (CTR).

For the second part of the study, human primary commercial osteoblasts (NHOst) purchased from LONZA (Morrisville, NC, USA) were expanded in appropriate commercial medium (Osteoblast growth medium: OGM, Lonza, Waskerville, MD, USA) containing 0.1% ascorbic acid, 0.1% gentamicin, and supplemented with 10% fetal bovine serum (FBS). The cultures were expanded at 37 °C in a 5% CO_2_/95% air-controlled atmosphere. At the same time, cultures of OCs were obtained as previously described.

The materials, placed in a culture insert (Millicel 0.4μm pore size, PCF 12 mm diameter, Millipore, Tulagreen Car-rigtwohill, Co. Cork, Ireland), were preconditioned with OGM for 2 h, then seeded with 5 × 10^4^ NHOst (1.5 × 10^5^/cm^2^). The cell seeding on materials was carried out on the 7th day of OC differentiation, separately in another well plate. The next day, when NHOst were properly attached to the scaffolds, the inserts were moved to the same wells with OC, thus assembling the co-cultures (Figure S1-Phase 2). As a control, NHOst were directly seeded in the culture inserts and assembled with OC adhering on bottom wells to verify cell viability and activity, regardless of material presence.

The co-culture medium was a mixture of osteogenic differentiation medium (ODM: OGM added with β-glycerophosphate and hydrocortisone, LONZA) and osteoclasts differentiation medium, prepared by maintaining a correct final concentration of each factor for both cellular components.

#### 2.3.2. Cell Viability

After the OC cultures and co-cultures (NHOst and OC) were set up, cell viability was quantified at 7 and 14 days. The materials seeded with NHOst were transferred to empty wells in order to appreciate the contribution of each cell type: viability of OC on the well bottom and viability of NHOst on the materials (see Phase 2 in Figure S1).

Alamar Blue™ dye (Invitrogen, Life Technologies Corporation, EUGENE, OR, USA) was added 1:10 *v/v* in culture medium separately to NHO_st_ and OC, both in the presence of materials and in CTR condition, and incubated for 4 h at 37 °C. This non-toxic reagent allows us to evaluate the cell activity on the same culture at different endpoints by the chemical reduction of an oxidized blue compound, which turns towards the purple color in the mitochondria of living cells. The fluorescent product was quantified by a Micro Plate reader (VICTOR X2030, Perkin Elmer, Milano, Italy) at 530 ex–590 em nm wavelengths and expressed as relative fluorescence units (RFU).

#### 2.3.3. OC Differentiation and Morphology

The evaluation of osteoclasts differentiation was performed, highlighting the presence of their typical marker- Tartrate-resistant acid phosphatase (TRAP)—by using a specific staining kit (TRAP kit, SIGMA, Buchs, Switzerland), following the manufacturer’s instructions. TRAP staining develops a purple color in positive cells, which typically show three or more nuclei, thus allowing the evaluation of osteoclastogenesis.

#### 2.3.4. Scaffold Colonization

The growth of the NHOst, both on the scaffolds and on the control well, was observed at the endpoint by the LIVE/DEAD™ assay (Invitrogen, Life Technologies), as previously reported [26]. Samples were visualized using an inverted microscope equipped with an epifluorescence setup (Eclipse TiU, NIKON Europe BV, NITAL SpA, Milano, Italy): excitation/emission setting of 488/530 nm to detect green fluorescence (live cells) and 530/580 nm to detect red fluorescence (dead cells).

#### 2.3.5. Gene Expression Analysis

Gene expression of the most common markers of OCs and NHOst was evaluated after 7 and 14 days of culture. Total RNA was extracted separately from NHOst seeded on the materials and OCs on the well bottom, as well as from the control cultures, using Trizol^®®^ reagent (AMBION by Life Technologies, Carlsbad, CA, USA) and Chloroform (Sigma Aldrich) until harvesting the aqueous phase. The procedure was continued using the commercial PureLinkTM RNeasy Mini Kit (AMBION), quantified by NANODROP spectrophotometer (NANODROP 2720, Thermal Cycler, Applied Biosystem, Waltham, MA, USA), and reverse transcribed with SuperScriptVILO cDNA Synthesis Kit (Life Technologies), following the manufacturer’s instructions. The obtained cDNA of each sample was diluted to the final concentration of 5 ng/μL, and semi-quantitative polymerase chain reaction (PCR) analysis was performed for each sample in duplicate in LightCycler 2.0 Instrument (Roche Diagnostics GmbH, Manheim, Germany) using QuantiTect SYBR Green PCR Kit (Qiagen, Hilden, Germany) and gene-specific primers (Table S1). The protocol included a denaturation cycle at 95 °C for 15″ and 25 to 40 cycles of amplification (95 °C for 15″, appropriate annealing temperature for each target for 20″, and 72 °C for 20″). After using the melting curve analysis to check for amplicon specificity, the threshold cycle was determined for each sample, and relative gene expression was calculated using the 2^−ΔΔCt^ method [27], with GAPDH as the reference gene and CTR samples as calibrators.

#### 2.3.6. Statistical Analysis

Statistical analysis was carried out by using R software version 4.2.1 (www.R-project.org, accessed on 23 June 2022) and package ‘dunn.test’ v.1.3.5 (https://CRAN.R-project.org/package=dunn.test , accessed on 27 October 2017). After checking the non-normal distribution (Shapiro Wilk test) and non-homogeneity of variance (Levene test) of the data, the Kruskal-Wallis χ^2^ test, followed by non-parametric pairwise multiple comparisons Dunn’s test, was applied to compare the results among groups (α level = 0.05).

## 3. Results and Discussion

### 3.1. Materials Synthesis and Characterization

DCPA and DCPD exhibit different crystalline structures, triclinic and monoclinic, respectively [28,29], which account for their different capability to host strontium ions [30,31].

The procedures utilized in this work to synthesize monetite and brushite in the presence of strontium provided single phases in both cases. In particular, the lattice constants of SrDCPA and SrDCPD reported in Table 2 are slightly enlarged in comparison to those of pure DCPA and DCPD. These parameters are in agreement with the strontium content of about 10 atom %, determined through ion chromatography (Table 2).

**Figure 1:**
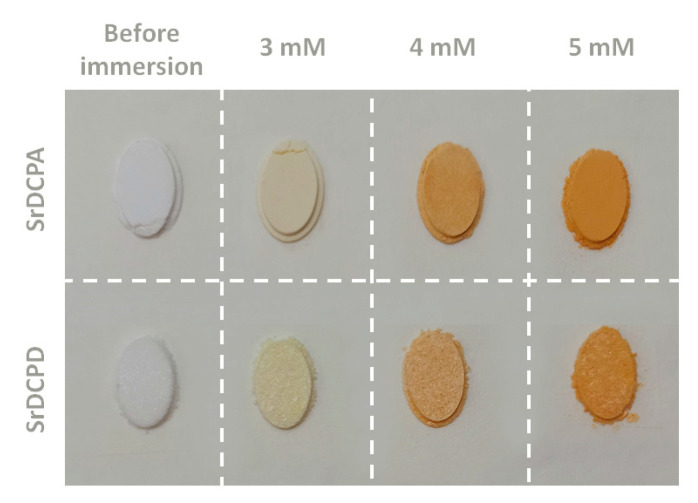
Photographic images of SrDCPA and SrDCPD crystalline powders before and after immersion in curcumin solutions at 3 mM, 4 mM, and 5 mM for 6 h.

The adsorption of curcumin on the phosphates was carried out at different times (6 and 72 h) through the immersion of SrDCPA or SrDCPD crystalline powders into EtOH/H_2_O solutions at increasing concentrations of the polyphenol up to 5 mM. After curcumin adsorption, the powders assume a yellow/orange color, which intensity increases with increasing time/concentration (Figure 1).

**Figure 2:**
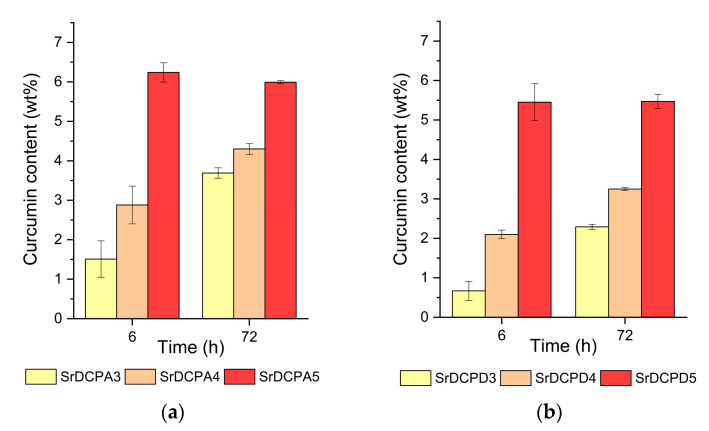
Curcumin content of SrDCPA (**a**) and SrDCPD samples (**b**) incubated in hydroalcoholic solutions at different curcumin concentrations and for different time periods.

Indeed, the curcumin content of the different samples increases with its concentration in a solution and with the exposure time up to about 6 wt%, as reported in Figure 2. Curcumin concentration in a solution of 5 mM allows it to reach the maximum amount of polyphenol content in the solid after a shorter exposure time of 6 h. Moreover, curcumin adsorption on SrDCPD samples is generally slightly smaller than that on SrDCPA samples.

**Figure 3:**
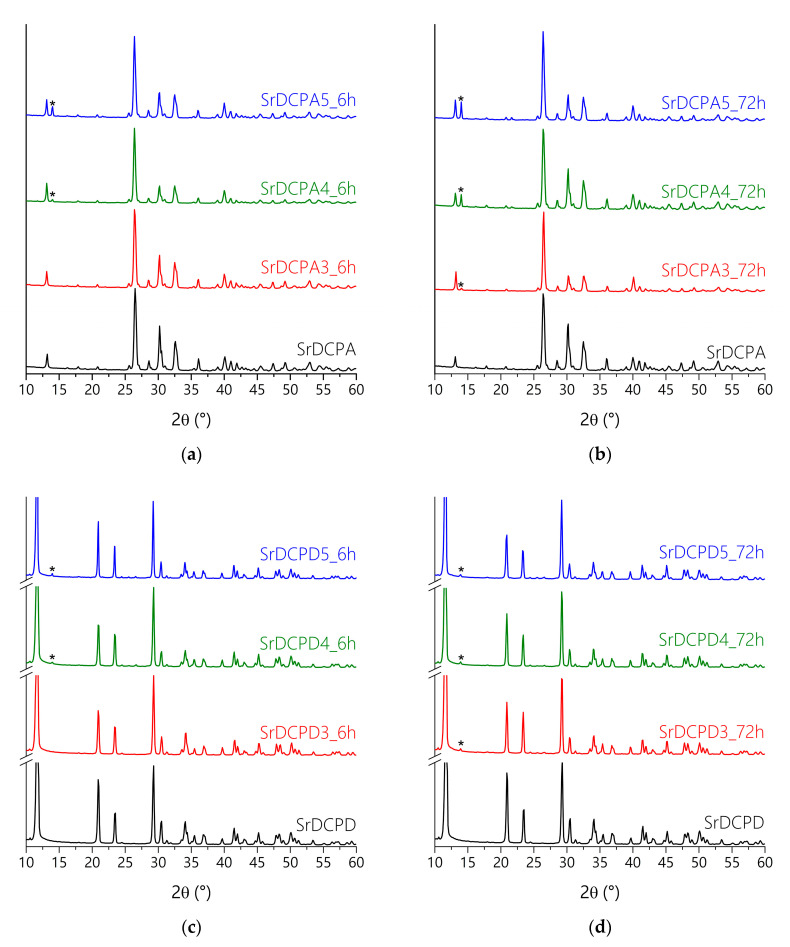
X-ray diffraction patterns of SrDCPA samples before and after incubation in curcumin solutions with different concentrations for 6 h (**a**) or 72 h (**b**), and of SrDCPD samples before and after incubation in curcumin solutions with different concentrations for 6 h (**c**) or 72 h (**d**). The peak indicated by (*) is due to the presence of crystalline curcumin.

The powder X-ray diffraction patterns of SrDCPA at different curcumin content reported in Figure 3a,b show the characteristic peaks of strontium substituted monetite. Moreover, all the patterns, except that of the sample submitted to interaction with 3 mM curcumin for 6 h, exhibit a small but appreciable reflection at 14°/2θ, which corresponds to the most intense reflection of crystalline curcumin [32]. The same reflection is visible in the powder X-ray diffraction patterns of SrDCPD submitted to interaction with curcumin (Figure 3c,d). The apparent smaller intensity of the reflection in the patterns of the brushite samples is mainly due to the higher relative intensity of the SrDCPD peaks in comparison to those of SrDCPA. The absence of the reflection characteristic of the polyphenol in the XRD patterns of SrDCPA and SrDCPD submitted to interaction with 3 mM curcumin for 6 h is in agreement with the relatively low presence of curcumin in these samples (Figure 2).

Further investigations were carried out on selected samples, namely on SrDCPA and SrDCPD submitted to interaction with curcumin 3 mM for 6 h (now onward labeled SA3 and SD3, respectively) and on SrDCPA and SrDCPD submitted to interaction with curcumin 5 mM for 72 h (now onward labeled SA5 and SD5, respectively). Similarly, pristine SrDCPA and SrDCPD are labeled SA0 and SD0 in the following.

**Figure 4:**
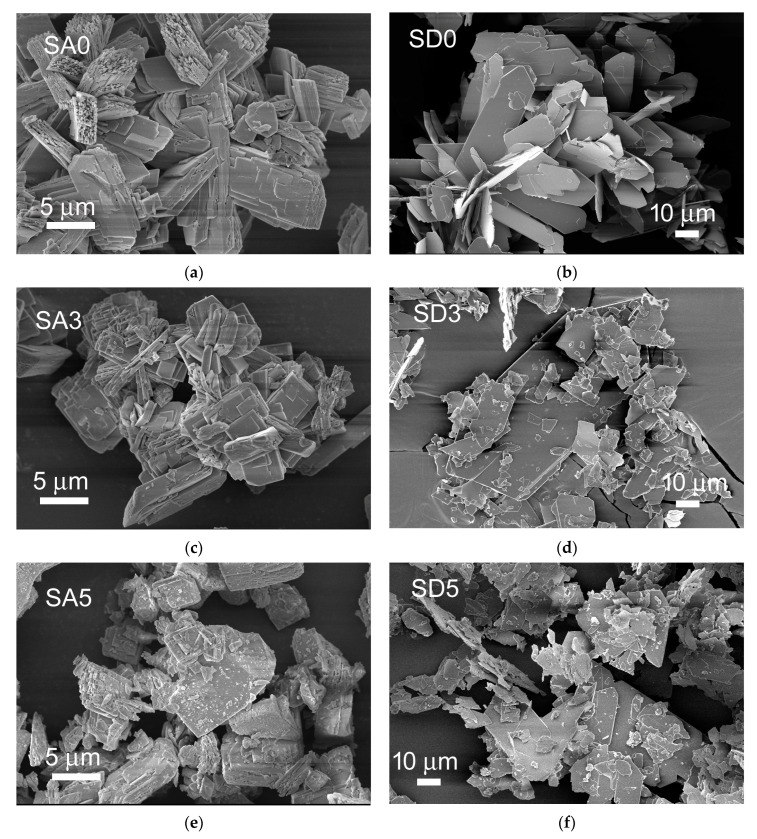
SEM images of calcium phosphates crystals before (**a**,**b**) and after incubation in curcumin solution for 6 h (**c**,**d**) and 72 h (**e**,**f**).

SEM images of the different samples are reported in Figure 4. The morphologies of monetite and brushite powders are quite different: SA0 is constituted of big, thick, and layered crystals, whereas SEM images of SD0 show even bigger crystals with a plate-like morphology: they exhibit wide (0k0) faces and tend to aggregate. After curcumin adsorption, both SA3 and SD3 show the presence of fragmented crystals, most likely due to the permanence of the samples in the solution. This effect is even more evident in the images of SA5 and SD5, where many significantly smaller crystals can be appreciated as well. In agreement with the XRD results, these very small crystals might be ascribed to curcumin precipitated onto the phosphate crystals.

**Figure 5:**
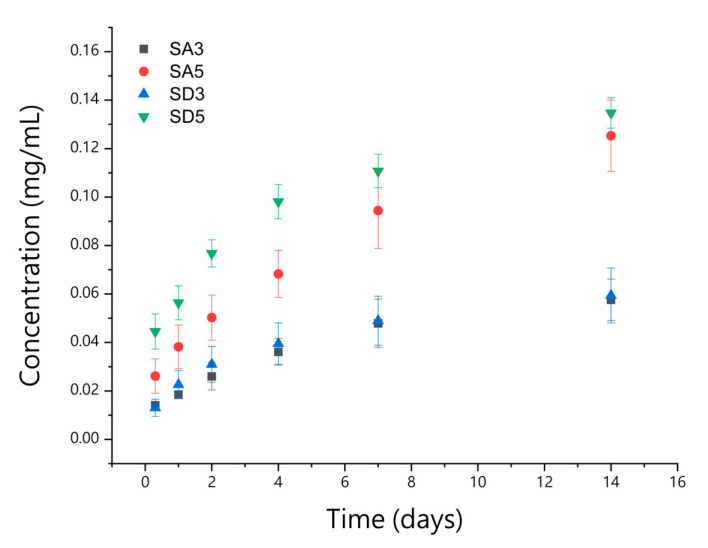
Curcumin release in Phosphate Buffer Solution up to 14 days.

The cumulative release of curcumin in PBS was monitored for up to 14 days. Figure 5 shows that curcumin cumulative release from the different samples increases with time. The maximum values of curcumin cumulative release from SA5 and SD5 are almost twice those from SA3 and SD3. In terms of percentages, release values do not exceed 10% of the initial curcumin content from SA5 and SD5, whereas higher percentages are released from SA3 and SD3 (Figure S2).

**Figure 6:**
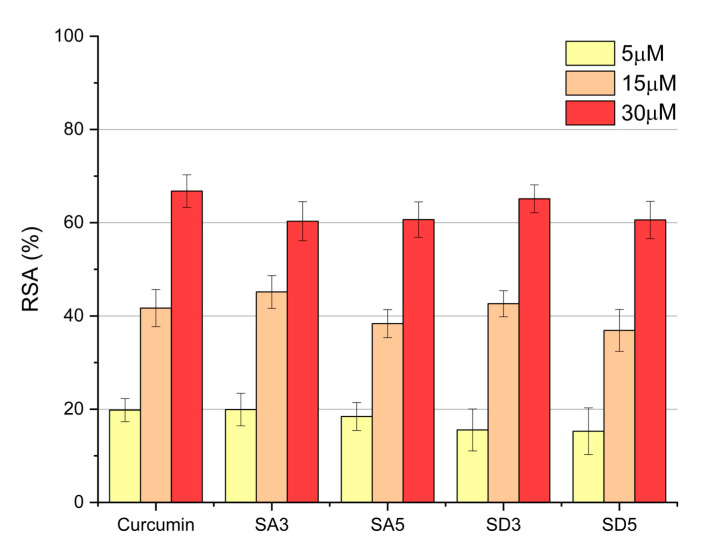
Antiradical activity, expressed as % RSA, of the different samples and pure curcumin toward DPPH•. Bars represent the mean ± SD of two independent measurements.

Curcumin is reported to exhibit anti-oxidant and anti-inflammatory properties thanks to its ability to quench reactive oxygen species [12,13,33]. In order to verify if the anti-oxidant properties are maintained after curcumin adsorption onto SrDCPA and SrDCPD, the radical scavenging activity (RSA) of the different samples was tested by means of the 1,1-diphenyl-2-picrylhydrazyl (DPPH•) assay [34,35]. The test is based on the reduction of the intensity of the DPPH• characteristic absorption band at 515 nm by the scavenging action of the anti-oxidant material. It was carried out on different amounts of samples in order to obtain the different concentrations of curcumin. The results show that the RSA of pure curcumin increases from about 20% to about 68% as the polyphenol concentration increases from 5 to 30 μM (Figure 6). The values recorded for SA3, SA5, SD3, and SD5 are similar to those of pure curcumin, indicating that the polyphenol maintains its activity after adsorption on the phosphates.

**Figure 7:**
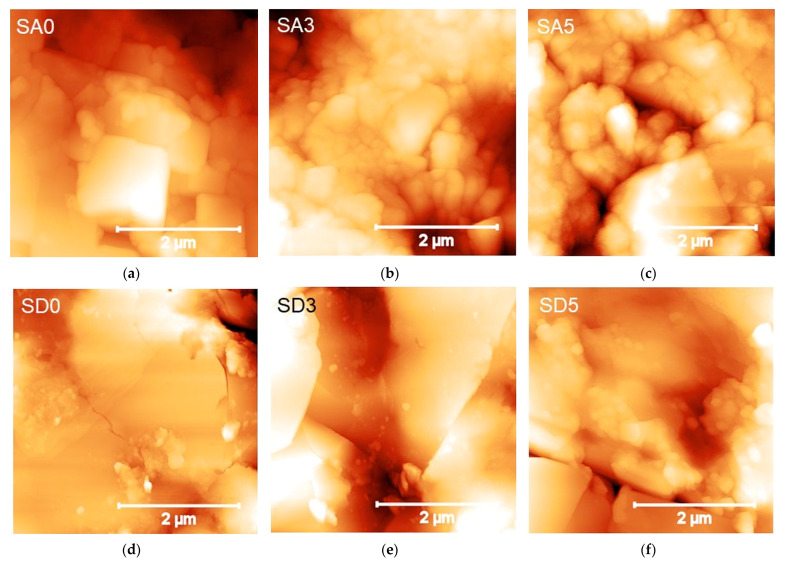
AFM images of 4 × 4 µm^2^ calcium phosphates crystals before (**a**,**d**) and after incubation in curcumin solution for 6 h (**b**,**e**) and 72 h (**c**,**f**).

On the sub-micrometer scale, AFM was used to investigate the evolution of the surface morphology of the substrates and possible changes in mechanical properties resulting from curcumin addition. Representative 4 × 4 µm^2^ AFM images of the two batches of SA0-SA3-SA5 and SD0-SD3-SD5 disks are shown (Figure 7). The micrographs confirmed the results of the SEM and XRD analysis as concerns the morphological and structural features of the corresponding powders (Figure 4). In particular, they show (i) the layered morphology of both SA0 and SD0 surfaces, with distinctly observable crystalline stacks, and (ii) the tendency to the aggregation of larger crystals, less pronounced in SA0 compared to SD0, as confirmed by the presence in the latter of several µm-large terraces with (0 k 0) orientation. As curcumin is adsorbed, the changes observed in the morphologies can be readily ascribed to the fragmentation process and the presence of small organic crystals.

Therefore, the mechanical properties of the SA and SD batches were measured via AFM-based nanoindentation, in order to probe the samples at length scales relevant to the biological environment and establish whether compositional and/or structural changes affect the mechanical response of the samples at an applied external load.

Table 3 reports the quantitative results of the nanoindentations operated within topographies (Figure 7). The different morphologies imply that the SA roughness is higher than SD, as confirmed by the R_S_ values (Table 3). The high (up to 40%) dispersion of the values are consistent with the observed complex topology and inhomogeneity of the surfaces. Small differences increase when the surfaces are mechanically probed. All SA and SD surfaces deform similarly (h_max_ = 25–30 nm) under the same indentation load (~6 µN). The measured deformation was essentially nearly elastic (0.5 < η_el_ < 1), however, η_el_ was lower for SA compared to SD. This slight but significant distinction between the two batches was independent of the addition of curcumin.

The elastic behavior of samples enables using Hertzian-like models to fit nanoindentation data [36]. The measured non-parametric distributions of indentation moduli for the SA and SD batches are reported in Figure S3. Remarkably, the median (50th percentile) values for SA0 and SD0 are similar (~16–17 GPa), in line with those reported for calcium phosphate cement and cortical bone [36,37]. The slight increase in depth observed with curcumin addition is expected to result in a decrease of moduli because of the h^−3/2^ dependence of the Hertz model. However, for SA3 and SD3, the stiffness did not change significantly compared to SA0 and SD0, while a reduction to about half the initial value was observed for the SA5 samples only.

By looking at the AFM results comprehensively, it can be suggested that the mechanical response of the SA and SD is similar, and the slight decrease with curcumin addition observed for SA5 is essentially due to the mechanical instability of grains compared to the plate-like and compact surface of SD, which is expected to exhibit a more elastic and stable response.

In the analyses above, some limitations should be considered, such as the probe sensitivity to the very first layers of samples: probing the free surface instead of the bulk may cause the detection of lower moduli compared to traditional nanoindentation measurement. However, η_el_, h_max_, and indentation moduli determined here depend on the geometry of the tip, so they should be seen as relative instead of absolute measurements of moduli. On the other hand, simultaneous monitoring of these quantities during indentations, as done in this work, helps in controlling measurement instabilities and ensures that indentations are effectively in the range of the model used to fit the data.

### 3.2. Biological Assays

**Figure 8:**
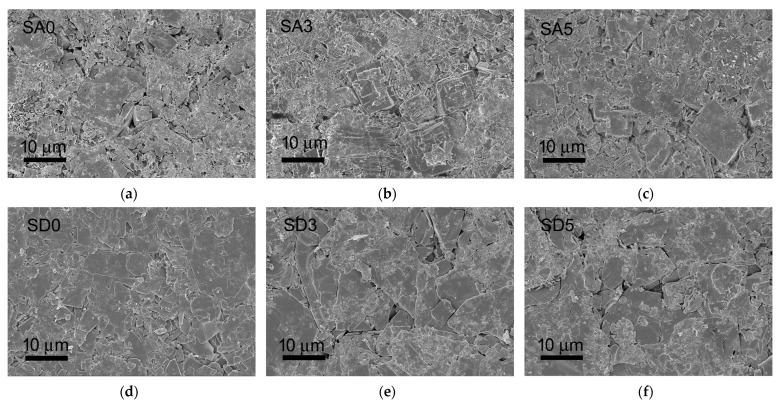
SEM images of the surface of disk-shaped samples obtained after pressing the powders into cylindrical molds: SA0 (**a**); SA3 (**b**); SA5 (**c**); SD0 (**d**); SD3 (**e**); SD5 (**f**).

In vitro tests were carried out on disk-shaped samples. The surfaces of the disks are flat and generally homogeneous, although few crystals displaying the characteristic morphology of the pristine SrDCPA and SrDCPD are still appreciable, as shown in Figure 8.

Recently, the influences on bone cells of pristine monetite (DCPA) and brushite (DCPD) were compared with their Sr-substituted forms [7]. The present study, which represents an evolution of the previous one for the addition of curcumin, opens the biological part with the observation of the OCs only and proceeds with that of both the main bone cells (NHOst and OCs) grown in co-culture. The presence of the Sr ion in all materials led to a focus primarily on the OCs’ behavior and, subsequently, on a more complex microenvironment closer to the physiological state.

Given the novelty of both the materials and the model based on the direct adhesion of cells to scaffolds, it is essential to proceed by first observing the fundamental parameters such as cell viability, activity, and morphology. Most studies on the interaction of curcumin with bone cells, including the very few on curcumin-loaded calcium phosphates, were carried out on powders suspended in the culture medium and not on disk-shaped samples [38,39,40,41].

**Figure 9:**
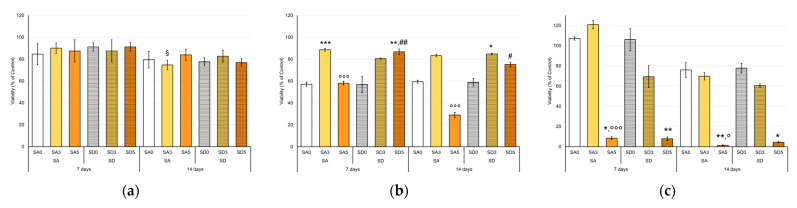
Cell viability (Alamar Blue Assay) of OCs culture (**a**), OCs co-cultured with NHOst (**b**), and NHOst co-cultured with OCs (**c**) with tested SA and SD disk-shaped samples, expressed as percentage variation from CTR cultures at 7 and 14 days (Mean ± SD, n = 4 duplicates). Dunn’s test (1 symbol, *p* < 0.05; 2 symbols, *p* < 0.005; 3 symbols, *p* < 0.0005): For each experimental time—Disk-shaped SA or SD samples with curcumin versus SA0 or SD0 (*); SA5 or SD5 versus SA3 or SD3, respectively (°); SD versus SA at each curcumin concentration (#); for each disk-shaped SA or SD samples with or without curcumin—14 days versus 7 days (§).

Cell viability, expressed as a percentage of CTR at each time point, displayed no relevant differences between OCs cultured in direct contact with SA and SD disk-shaped samples. All values are ≥ 70%, therefore, no material resulted as cytotoxic for OCs (Figure 9a), and the values lower than the controls are likely due to the presence of Sr [42].

OCs showed different behavior in the NHOst/OC co-cultures. Viability values were below the 60% level at 7 days of co-culture for SA0 (57%), SA5 (58%), and SD0 (57%), while SA3 (89%), SD3 (80%), and SD5 (87%) showed significantly higher values (Figure 9b), similar to what was observed for OCs directly seeded on the same scaffolds. At 14 days of co-culture, the OCs viability values remained substantially like those at 7 days for each material, with the exception of SD5, whereas the SA5 samples exhibited a further deterioration in viability (29%). From these viability data, it could be hypothesized that curcumin (i) in its intermediate concentration counterbalances the action of Sr on OCs for both SA and SD samples and (ii) in its higher concentration for SD-based samples.

The materials SA0 (108%), SD0 (106%), and SA3 (121%) showed viability values of NHOst even higher than CTR (considered 100%) at 7 days of co-culture with the OCs (Figure 9c). At 14 days, the NHOst viability values decreased for all the materials, probably due to the achieved hyper-confluence. However, viabilities on SA0 (76%), SD0 (77%), and SA3 (70%) resulted in still the highest ones, followed by SD3 (60%). On the other hand, it is evident that the highest curcumin content severely compromises NHOst viability, which decreases from already very low values at 7 days (10%) to values close to zero at 14 days. This is confirmed by the images of fluorescently labeled cells (Figure S4) and is in agreement with Moran et al., who defined a cut-off of curcumin concentration in the microenvironment of culture [43]. For this reason, the second part of the study on co-culture was focused on SA0, SA3, and SD0.

In order to further investigate the response of main bone cells to monetite or brushite enriched with curcumin, the expression of some genes was evaluated. For OC, the expression of Acid Phosphatase 5, Tartrate Resistant (*ACP5*), and Cathepsin K (*CTSK*) was quantified both in single culture and in OCs differentiated in the presence of NHOst.

**Figure 10:**
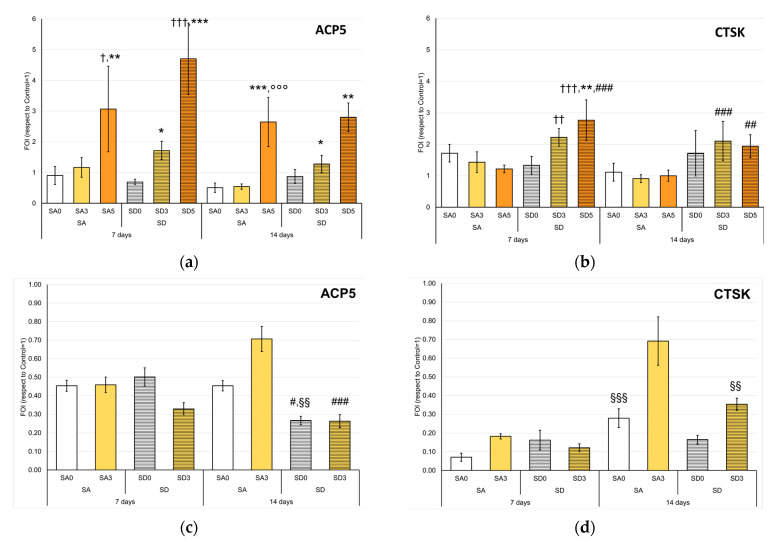
Expression of the *ACP5* (**a,c**), *CTSK* (**b,d**) genes in OC (**a,b**), and OC co-cultured with NHOst (**c,d**) after 7 and 14 days (Mean ± SD, n = 4 duplicates). Dunn’s test (1 symbol, *p* < 0.05; 2 symbols, *p* < 0.005; 3 symbols, *p* < 0.0005): Each condition versus Control (†); or each experimental time—Disk-shaped SA or SD samples with curcumin versus SA0 or SD0 (*); SA5 or SD5 versus SA3 or SD3, respectively (°); SD versus SA at each curcumin concentration (#); For each disk-shaped SA or SD samples with or without curcumin—14 days versus 7 days (§).

In single culture, the expression of *ACP5*, gene coding for TRAP, showed to be more pronounced in the samples at the highest concentrations of curcumin than CTR, SA0, and SD0, both at 7 and 14 days. Overall, the increase in curcumin content in the materials corresponded to a significant increase in *ACP5* expression, with the greatest differences between SA5 and SA0, as well as between SD3, SD5, and SD0 (Figure 10a). Interestingly, TRAP staining showed an increasing number of small and less differentiated OCs with the increase of curcumin amount in the materials, as if the differentiation process was somehow inhibited by other post-transcriptional factors. Li et al. demonstrated the role of miRNA-365 on the MMP9 downregulation in osteoporotic mice treated with curcumin, as well as a decreased TRAP-positive area in histological sections of tibias [44]. In a similar model, the study of Chen Z.et al. confirmed a positive effect of curcumin on bone microarchitecture and a partial recovery of WNT signaling inhibition in osteoporotic mice. [45]. Furthermore, a population characterized by very large and multinucleated cells was appreciated, particularly in the presence of SA3 and SD3 samples (Figure S5 and S6). When co-cultured with NHOst, no significant differences were observed among the samples at 7 days, while at 14 days, SD samples showed a lower expression of *ACP5* than SA. It is important to note that in the presence of NHOst, the expression of this gene was further reduced and was always inferior to one, which represents the value of the CTR (Figure 10c). TRAP staining images confirmed this observation (Figure S7), and mostly the aspect of undifferentiated cells on SA5 and SD5 was even more evident in co-culture. The cell count of undifferentiated and differentiated osteoclasts performed after 14 days in single culture and co-culture showed an increase in undifferentiated cells proportional to the curcumin content, especially in SD5 (Table S2). Curcumin could inhibit osteoclastogenesis by acting through the suppression of ROS generation, proportionally to its concentration [46].

*CTSK* is a gene encoding a protein involved in bone remodeling activity; its secretion in the extracellular compartment, between OCs and bone surface, induces organic matrix degradation. Figure 10b allows us to appreciate how the presence of curcumin has a different impact on the expression of *CTSK* based on the substrate: SD samples showed a higher expression of *CTSK* than SA samples. The first time point showed a more regular trend as a function of curcumin content: in line with previous studies, which attribute curcumin to the role of osteoclastogenesis suppressor, [47] *CTSK* expression decreased in the SA group by adding curcumin, the opposite trend was observed for SD group. Again, however, the gene expression decreased dramatically in the presence of NHOst, although it showed some increase over time (Figure 10d).

**Figure 11:**
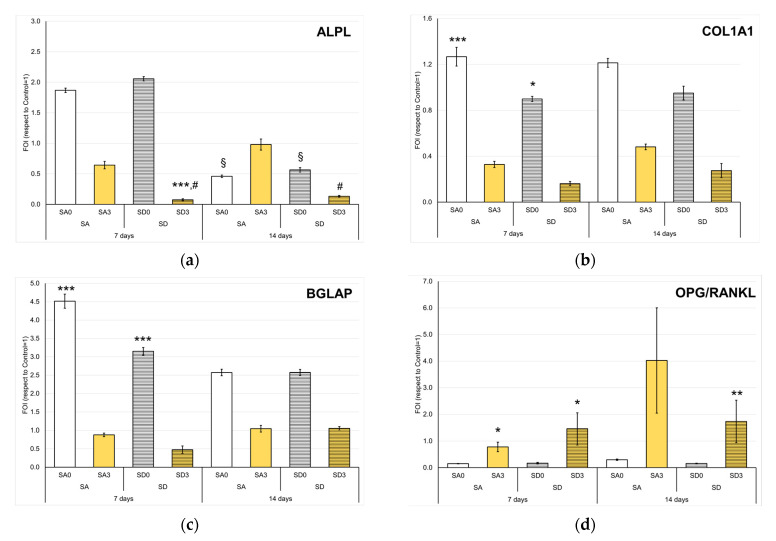
Expression of the *ALPL* (**a**), *COL1A1* (**b**), *BGLAP* (**c**), and OPG/RANKL (**d**) genes in NHOst co-cultured with OCs after 7 and 14 days (Mean ± SD, n = 4 duplicates). Dunn’s test (1 symbol, *p* < 0.05; 2 symbols, *p* < 0.005; 3 symbols, *p* < 0.0005): For each experimental time—Disk-shaped SA or SD samples with curcumin versus SA0 or SD0 (*); SD versus SA at each curcumin concentration (#); for each disk-shaped SA or SD samples with or without curcumin—14 days versus 7 days (§).

Monetite and brushite confirmed their osteoinductive role on NHOst, showing higher values than CTR in the expression of *ALPL*, *COL1A1*, and osteocalcin (*BGLAP*) [19]. Conversely, in the presence of curcumin, these genes were less expressed, as if the cells were in a less differentiated state. (Figure 11a–c). Over time, in fact, the values increased for SA3 and SD3 (even without reaching significance) and decreased for the other samples.

Interestingly, the OPG/RANKL ratio was always significantly higher in SA3 and SD3 than in SA0 and SD0, respectively (Figure 11d). A similar result was reported for cultures of osteoblasts treated with curcumin supplied in liposomes [48]. Furthermore, a positive effect of curcumin was previously reported on the ratio of OPG/RANKL released in the culture supernatant of fibroblasts [49]. In the delicate balance between osteosynthesis and bone resorption, this could be the effective contribution of curcumin. The presence of Sr would exert an inhibiting role on bone resorption by decreasing RANKL and increasing OPG levels [50], and curcumin could support this mechanism [51].

## 4. Conclusions

The results of this work demonstrate that strontium-substituted monetite, as well as strontium-substituted brushite, can be further functionalized with curcumin, providing materials with very good radical scavenging activity and a sustained release in PBS. The polyphenol can be loaded onto the substrates from hydroalcoholic solution up to about 5–6 wt%, with slight differences between the two substrates, most likely related to their different morphology. No significant modification of the structure, morphology, and stiffness of SrDCPA and SrDCPD occurs because of curcumin adsorption.

The results of in vitro tests indicate that for the main bone cells (osteoblasts and osteoclasts), both the content of curcumin and the type of dicalcium phosphate (monetite or brushite) seem to be relevant: an increasing amount of curcumin in the biomaterial composition caused different responses by NHOst and OCs, and different substrates containing the same amount of curcumin sometimes elicited different biological reactions. The highest content of curcumin provoked negative effects on osteoblast viability. SA5 and SD5 also stimulated the expression of the OC typical genes and strongly altered the morphology and differentiation of these cells. On the other hand, a lower curcumin content seemed to provide osteoclasts with adequate stimulus, in line with what was observed on Sr-substituted monetite and brushite. The activity of the osteoclasts, albeit moderate, is important in pathologies such as osteoporosis to maintain good physiology of bone tissue and to allow the resorption of biomaterials based on calcium phosphates.

## Figures and Tables

**Table 1:** List of the samples submitted to all tests and label correspondence with preliminary tests.

Description of the Sample	Labels Used in Preliminary Tests	Labels Used in Complete Tests
Strontium-substituted DCPA (before loading-sample without curcumin)	SrDCPA	SA0
Strontium-substituted DCPD (before loading-sample without curcumin)	SrDCPD	SD0
SrDCPA in 3 mM curcumin solution for 6 h	SrDCPA3_6 h *	SA3
SrDCPD in 3 mM curcumin solution for 6 h	SrDCPD3_6 h *	SD3
SrDCPA in 5 mM curcumin solution for 72 h	SrDCPA5_72 h *	SA5
SrDCPD in 5 mM curcumin solution for 72 h	SrDCPD5_72 h *	SD5

* SrDCPAx_yh, or SrDCPDx_yh, where x is the concentration of curcumin solution and y is the time period of incubation.

**Table 2:** Crystalline cell parameters and Sr content in Strontium-substituted DCPA and DCPD.

Sample	a (Å)	b (Å)	c (Å)	α (°)	β (°)	γ (°)	Sr Content (at%)
SrDCPA	6.936 (5)	6.644 (4)	7.015 (5)	96.1	104.1	88.5	11.2
SrDCPD	6.394 (3)	15.239 (3)	5.830 (4)	90	118.4	90	9.4

**Table 3:** Results of the nanomechanical analysis by AFM. The root-mean-square roughness R_s_, the maximum indentation depth h_max_, and elasticity index η_el_ are reported.

	SA0	SA3	SA5	SD0	SD3	SD5
R_s_ (nm)	172 ± 58	120 ± 30	149 ± 22	86 ± 35	87 ± 13	106 ± 11
h_max_ (nm)	25.5 ± 6.6	27.3 ± 7.9	31.7 ± 9.2	26.6 ± 6.6	28.0 ± 10.2	30.5 ± 10.5
η_el_	0.654 ± 0.127	0.572 ± 0.188	0.579 ± 0.173	0.730 ± 0.106	0.778 ± 0.094	0.616 ± 0.158

## Data Availability

The data presented in this study are available on request from the corresponding author.

## Supplementary Materials

The following supporting information can be downloaded at: https://www.mdpi.com/article/10.3390/pharmaceutics15051344/s1, Figure S1: experimental set-up for in vitro tests; Figure S2: curcumin release in phosphate buffer solution; Figure S3: Non-parametric distributions of the indentation moduli calculated by Hertz model; Figure S4: Live and Dead fluorescent labeling of NHOst; Figure S5: TRAP staining at endpoint (20×)—OC in single culture; Figure S6: TRAP staining at endpoint (10×)—OC in single culture; Figure S7: TRAP staining at endpoint (10×). OC in co-culture; Table S1: details of primers used for gene expression analysis; Table S2: Differentiated and undifferentiated cell count.
