# Supplementary material for: Anti-Oxidant Multi-Functionalized Materials: Strontium-Substituted Monetite and Brushite as Delivery Systems for Curcumin
Source: Pharmaceutics. 2023 Apr 27;15(5):1344. doi: 10.3390/pharmaceutics15051344 (PMC10224121; doi:10.3390/pharmaceutics15051344)
Supplement: Supplementary file 1 [file pharmaceutics-15-01344-s001.zip › pharmaceutics-2269520-supplementary.pdf]

*Supplementary Materials*

# Anti-oxidant multi-functionalized materials: strontium-substituted monetite and brushite as delivery systems for curcumin

Francesca Silingardi, Stefania Pagani, Alessandro Gambardella, Gianluca Giavaresi, Adriana Bigi, and Elisa Boanini

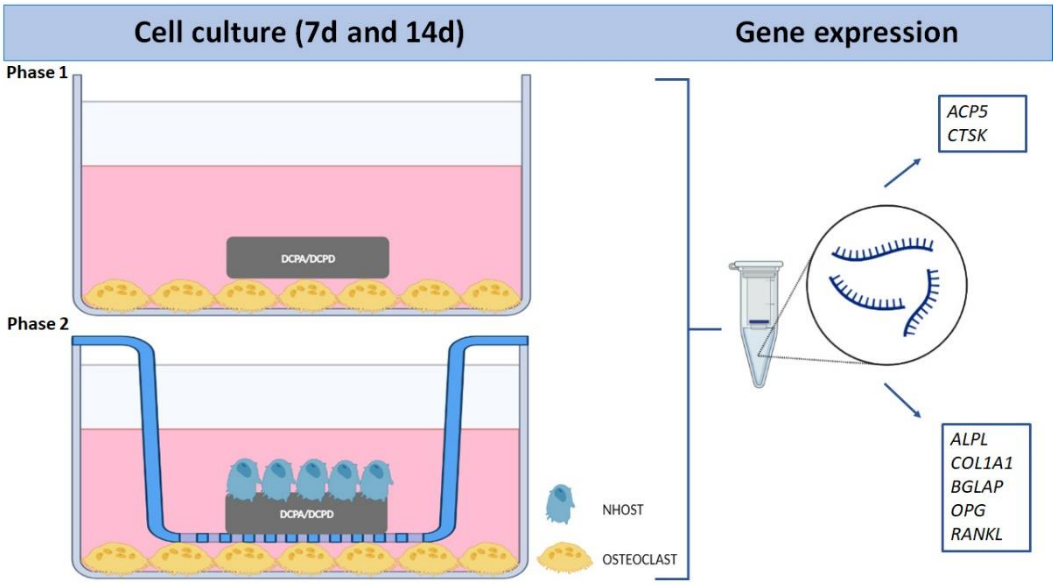


**Figure S1 –** experimental set-up for in vitro tests

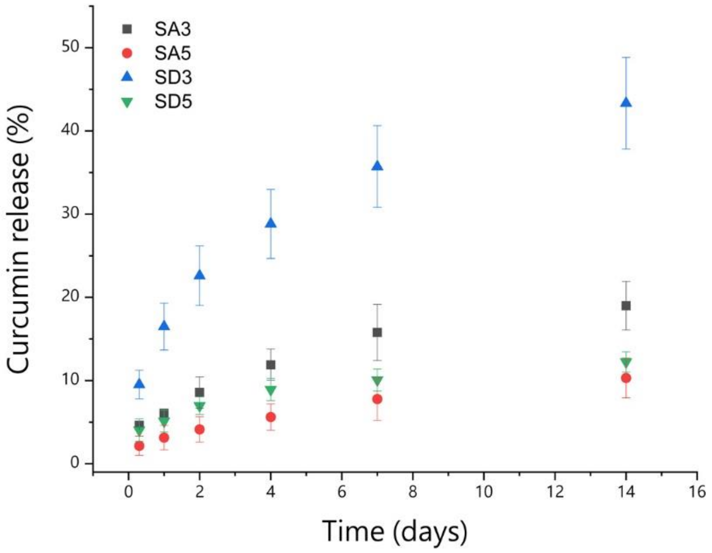


**Figure S2** – Curcumin release in Phosphate Buffer Solution up to 14 days.

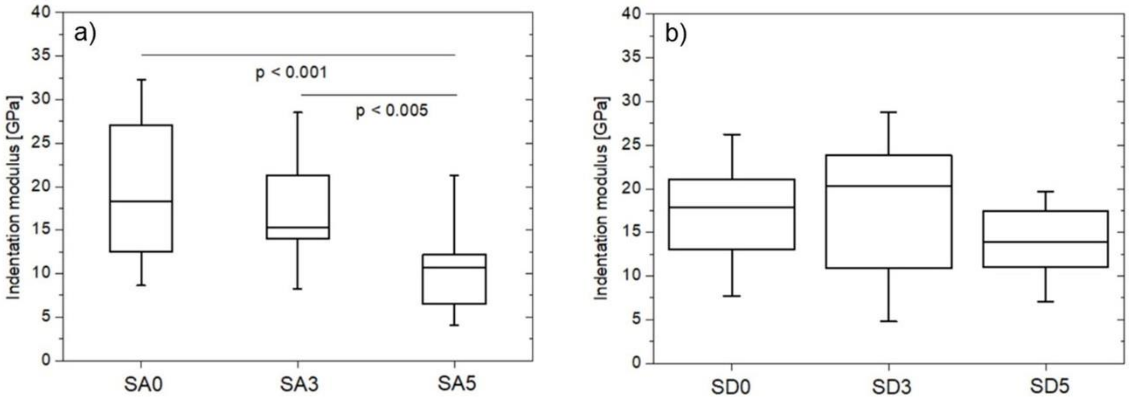


**Figure S3** –Non-parametric distributions of the indentation moduli calculated by Hertz model on the SA (a) and SD (b) batches. Only statistically meaningful ($p < 0.05$) differences are evidenced.

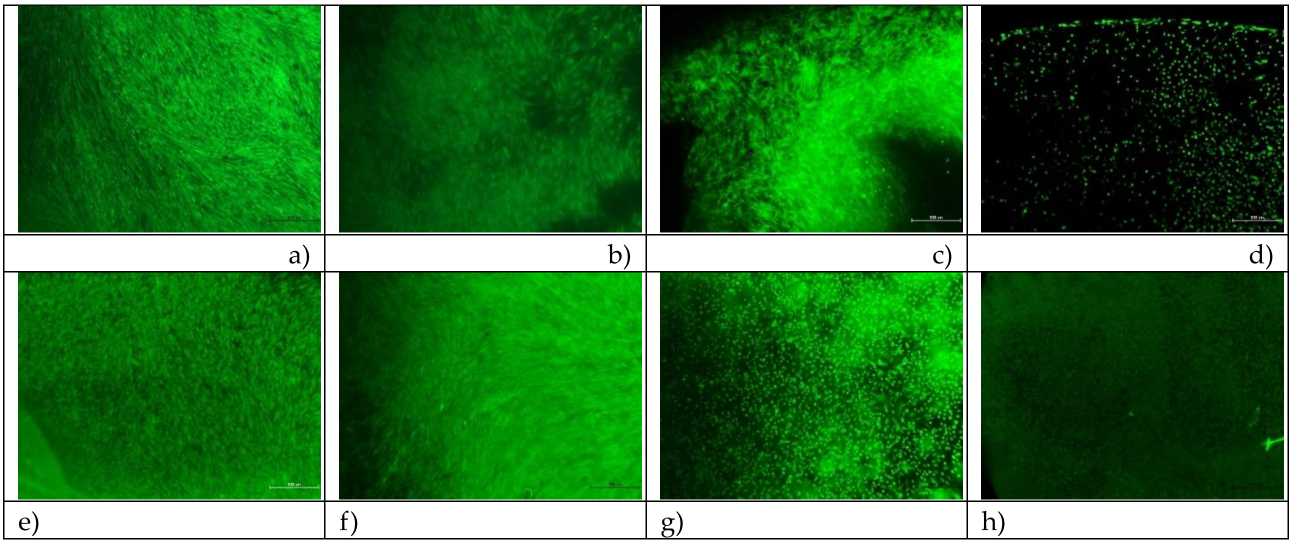


**Figure S4** – Live and Dead fluorescent labelling of NHOst after 2 weeks of culture (4x): Cells seeded on well bottom as single culture of osteoblast (a), and in coculture condition (CTR) (e); NHOst on SA0, SA3, SA5 (b, c, d), on SD0, SD3, SD5 (f, g, h) in co-culture condition. Scale bars: 500 μm.

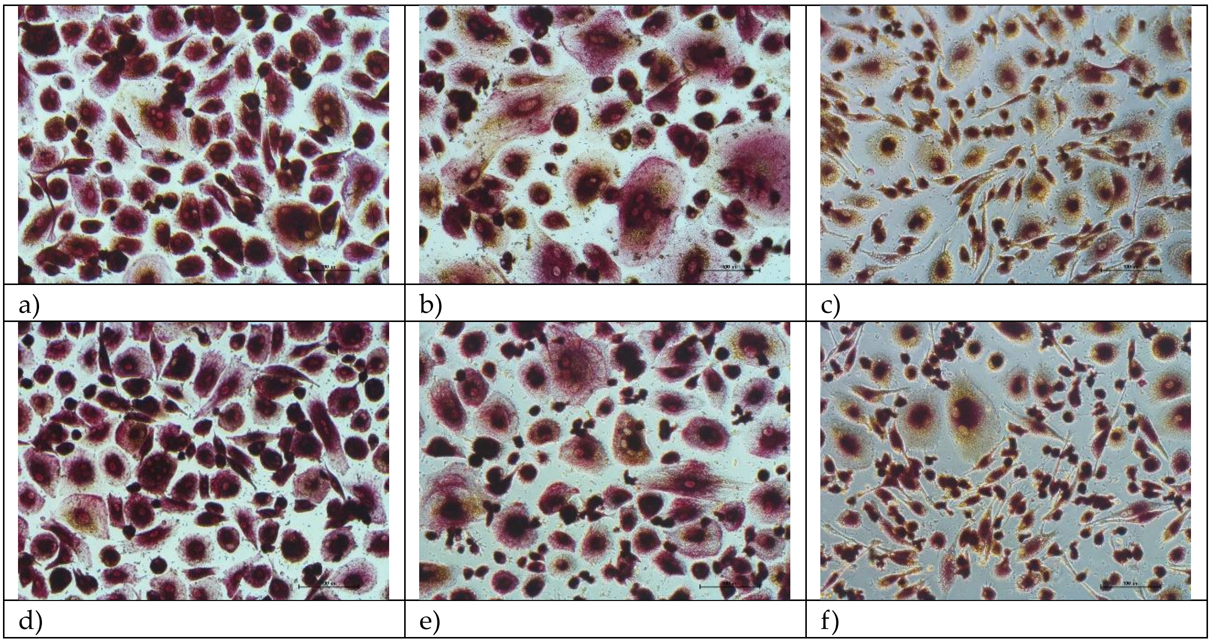


**Figure S5** – TRAP staining at endpoint (20x). OC in single culture in the presence of SA0, SA3, SA5 (a, b, c), and SD0, SD3, SD5 (d, e, f). Scale bars: 500 μm.

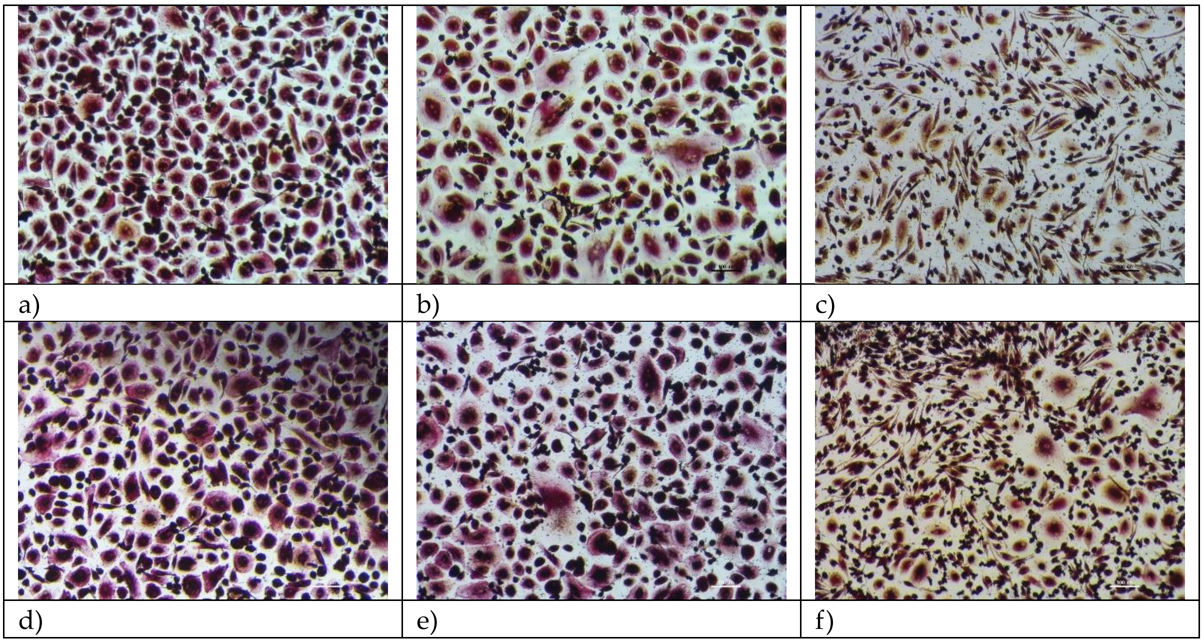


**Figure S6** – TRAP staining at endpoint (10x). OC in single culture in the presence of SA0, SA3, SA5 (a, b, c), and SD0, SD3, SD5 (d, e, f). Scale bars: 100 μm.

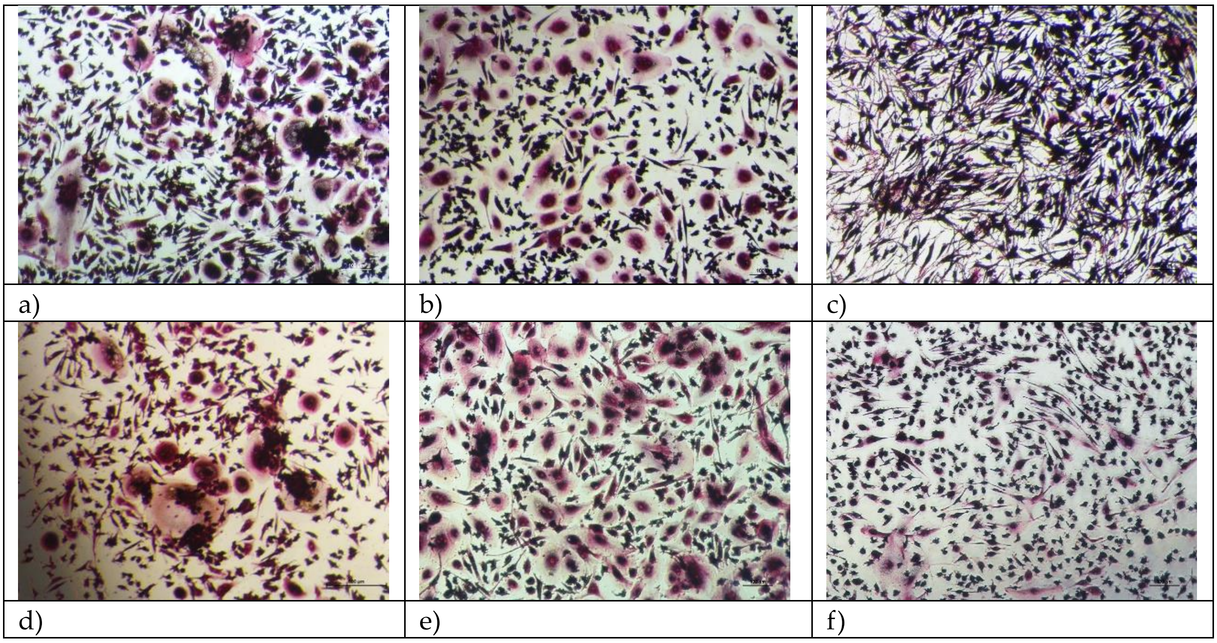


**Figure S7** – TRAP staining at endpoint (10x). OC in co-culture in the presence of SA0, SA3, SA5 (a, b, c), SD0, SD3, SD5 (d, e, f). Scale bars: 100 µm

**Table S1 –** Details of primers used for gene expression analysis.

| Gene | Primer forward | Primer reverse | Amplicon Lenght | Annealing Temperature |
|---|---|---|---|---|
| ***GAPDH*** | 5’-TGGTATCGTGGAAGGACTCA-3’ | 5’-GCAGGGATGATGTTCTGGA-3’ | 123 bp | 56°C |
| ***COL1A1*** | QuantiTect Primer Assay (Qiagen) Hs_COL1A1_1_SG | | 118 bp | 55°C' |
| ***ALPL*** | QuantiTect Primer Assay (Qiagen) Hs_ALPL_1_SG | | 110 bp | 55°C' |
| ***BGLAP*** | QuantiTect Primer Assay (Qiagen) Hs_BGLAP_1_SG | | 90 bp | 55°C' |
| ***TNFRSF11B (OPG)*** | QuantiTect Primer Assay (Qiagen) Hs_TNFRSF11B_1_SG | | 107 bp | 55°C' |
| ***TNFSF11 (RANKL)*** | 5’-TGAGATGAGCAAAAGGCTGAG-3’ | 5’-AGGAGCTGTGCAAAAGGAAT-3’ | 134 bp | 60°C' |
| ***CTSK*** | 5’-CAGACAACAGATTTCCATCAGC-3’ | 5’-CTTCTTCCATAGCTCCCAGTG-3’ | 118 bp | 60°C' |
| ***ACP5*** | 5’-GAAGCGCAGATAGCCGTT-3’ | 5’-GGTCACTGCCTACCTGTG-3’ | 148 bp | 60°C' |

**Table S2 –** Differentiated and undifferentiated cell count. Count values are indicated in the first line (N), while the percentage of each cell type (undifferentiated and differentiated) is indicated in the second line (%).

| | **OSTEOCLASTS in single culture** | | | | | | | | | | | |
|---|---|---|---|---|---|---|---|---|---|---|---|---|
| | **SA0** | | **SA3** | | **SA5** | | **SD0** | | **SD3** | | **SD5** | |
| | Undiff | Differ | Undiff | Differ | Undiff | Differ | Undiff | Differ | Undiff | Differ | Undiff | Differ |
| N | 195 | 159 | 146 | 91 | 237 | 73 | 167 | 152 | 173 | 82 | 236 | 64 |
| % | 55 | 45 | 62 | 38 | 76 | 24 | 52 | 48 | 68 | 32 | 79 | 21 |

| | **OSTEOCLASTS in co-culture** | | | | | | | | | | | |
|---|---|---|---|---|---|---|---|---|---|---|---|---|
| | **SA0** | | **SA3** | | **SA5** | | **SD0** | | **SD3** | | **SD5** | |
| | Undiff | Differ | Undiff | Differ | Undiff | Differ | Undiff | Differ | Undiff | Differ | Undiff | Differ |
| N | 320 | 55 | 296 | 82 | 350 | 16 | 409 | 50 | 270 | 50 | 593 | 18 |
| % | 85 | 15 | 78 | 22 | 96 | 4 | 89 | 11 | 84 | 16 | 97 | 3 |

**Materials and Methods**

**Cell cultures**

The present study was developed in 2 phases. Firstly, these biomaterials based on monetite or brushite partially substituted with strontium and doped with curcumin were tested by cultures of human primary osteoclast (OC). Subsequently, the biomaterials were tested by co-cultures consisting in OCs and human primary osteoblasts (NHOst).

OCs were obtained by mononuclear cells isolated from peripheral human buffy coat of a healthy adult male donor (Ethic Comitee -CE AVEC- approval n. 191/2019/Sper/IOR, 04/19), by density gradient centrifugation (Ficoll Histopaque 1077, Sigma Aldrich, St. Louis, MO, USA). Briefly, a volume of buffy coat, diluted 1:1 with pre-warmed PBS, was carefully layered on Ficoll according to 2:1 ratio, and centrifuged at 600g, room temperature, for 30 min. The obtained cells were washed twice in PBS, counted and seeded on 24-well plate bottom at the density of $1x10^6$ cells/$cm^2$ in Dulbecco's modified Eagle medium (DMEM) supplemented with 10% FCS and antibiotics (100 U/ml penicillin, 100 μg/ml streptomycin). After 24 hrs, non-adherent cells were removed and the growth medium replaced with DMEM added with 25 ng/ml macrophage colony-stimulating factor (M-CSF), 30 ng/ml receptor activator for NFkB factor ligand (RANKL), and $10^{-7}$ M parathyroid hormone (PTH) (Peprotech, Rocky Hill, NJ, USA). The cells were monitored frequently under microscope about adhesion and morphology, and allowed to differentiate into OCs in 1 week. After this time, each material was placed on the OC layer and the cell cultured evaluated at 7 and 14 days. OCs without materials were used as control (CTR).

For the second part of the study, human primary commercial osteoblasts (NHOst) purchased from LONZA (Morrisville, NC, USA) were expanded in appropriate commercial medium (Osteoblast growth medium: OGM, Lonza, Waskerville, MD, USA) containing 0.1% ascorbic acid, 0.1% gentamicin, and supplemented with 10% fetal bovine serum (FBS). The cultures were expanded at 37 °C in 5% $CO_2$/95% air-controlled atmosphere. At the same time, cultures of OCs were obtained as previously described.

The materials, placed in culture insert (Millicel 0.4μm pore size, PCF 12mm diameter, Millipore, Tulagreen Car-rigtwohill, Co. Cork, Ireland), were before preconditioned with OGM for 2 hrs, then seeded with $5x10^4$ NHOst ($1.5x10^5/cm^2$). The cell seeding on materials was carried out on the $7^{th}$ day of OC differentiation, separately in another well plate. The next day, when NHOst were properly attached to the scaffolds, the inserts were moved to the same wells with OC, thus assembling the co-cultures (Figure S1). As control, NHOst were directly seeded in the culture inserts and assembled with OC adhering on bottom wells, to verify cell viability and activity, regardless of material presence.

The co-cultures medium was a mixture of osteogenic differentiation medium (ODM: OGM additioned with β-glycerophosphate and hydrocortisone, LONZA) and osteoclasts differentiation medium, prepared by maintaining a correct final concentration of each factor for both cellular components.

**Scaffold colonization**

The growth of the NHOst, both on the scaffolds and on the control well, was observed at endpoint by the LIVE/DEAD™ assay (Invitrogen, Life Technologies): a mixture of calcein-AM and ethidium bromide, according to the manufacturer’s instructions. Intracellular esterases of live cells are able to convert calcein-AM into green fluorescent calcein, while the binding between DNA and ethidium, entering the dead cells through the damaged membranes, is able to develop red fluorescence. Samples were visualized using an inverted microscope equipped with an epifluorescence setup (Eclipse TiU, NIKON Europe BV, NITAL SpA, Milano, Italy): excitation/emission setting of 488/530 nm to detect green fluorescence (live cells) and 530/580 nm to detect red fluorescence (dead cells).
